# Clinical utility of metagenomic next-generation sequencing in infants with severe infections

**DOI:** 10.3389/fmicb.2026.1842600

**Published:** 2026-07-16

**Authors:** Yuanyuan Gao, Yuhan Huang, Wenmei Li, Yan Huang, Xianhong Zhao, Chu Chu, Xin Zhang, Jie Chen, Yunzhong Wang, Yang Li, Haifeng Geng

**Affiliations:** 1 Department of Clinical Laboratory, Children’s Hospital of Soochow University, Suzhou, Jiangsu, China; 2 Department of Neonatology, Children’s Hospital of Soochow University, Suzhou, China; 3 Department of Pharmacy, Children’s Hospital of Soochow University, Suzhou, China; 4 Department of Respiratory Medicine, Children’s Hospital of Soochow University, Suzhou, China; 5 Department of Infectious Diseases, Children’s Hospital of Soochow University, Suzhou, China

**Keywords:** clinical characteristics, infants, metagenomic next-generation sequencing, pathogenic microorganisms, severe infection

## Abstract

**Objective:**

This study aimed to compare pathogen detection rates between metagenomic next-generation sequencing (mNGS) and conventional microbiological culture in critically ill infants younger than 1 year of age, and to investigate the associations between mNGS positivity and clinical laboratory parameters.

**Methods:**

We conducted a single-centre retrospective study including infants with severe infections admitted to the Children’s Hospital of Soochow University between 1 January 2023 and 31 December 2025, who underwent both mNGS and conventional culture testing. Patients were classified into mNGS-positive and mNGS-negative groups, and clinical characteristics and laboratory findings were compared between groups. Candidate predictors of mNGS positivity were identified using least absolute shrinkage and selection operator (LASSO) regression, followed by multivariable logistic regression analysis. The predictive performance of key variables was evaluated using receiver operating characteristic (ROC) curve analysis.

**Results:**

A total of 105 infants and 153 biological specimens were included. The overall mNGS positivity rate, as well as positivity rates across all specimen types except cerebrospinal fluid, were significantly higher than those of conventional culture (*p* < 0.05). LASSO regression identified eosinophil percentage, mean corpuscular haemoglobin (MCH), procalcitonin (PCT), cholinesterase, and serum calcium as candidate predictors of mNGS positivity. Multivariable logistic regression revealed that MCH (OR = 0.755, 95%CI: 0.657–0.869), cholinesterase (OR = 0.999, 95%CI: 0.999–1.000), and PCT (OR = 1.180, 95%CI: 1.050–1.320) were independently associated with mNGS positivity. ROC analysis demonstrated that MCH, cholinesterase, and PCT individually showed moderate discriminatory performance, whereas a combined model incorporating all three variables achieved substantially improved predictive performance (AUC = 0.842, 95%CI: 0.758–0.912), with a sensitivity of 87.2% and specificity of 81.6%.

**Conclusion:**

mNGS demonstrated superior pathogen detection compared with conventional culture in critically ill infants. MCH, cholinesterase, and PCT were independently associated with mNGS positivity, and a combined multi-marker model substantially improved the prediction of mNGS-positive cases.

## 1 Introduction

Severe infections remain a major cause of mortality and serious complications in infants and young children. These infections are characterised by diverse pathogens, complex disease courses, and often atypical clinical presentations, posing significant challenges for early diagnosis and targeted therapy. Conventional diagnostic methods have limited sensitivity for certain pathogens in critically ill patients, which may delay timely antimicrobial intervention and impede individualised treatment adjustments (Zhang et al., 2022). In recent years, metagenomic next-generation sequencing (mNGS) has emerged as an unbiased, high-throughput approach for pathogen detection and has gained increasing clinical interest in the diagnosis of infectious diseases. By enabling rapid sequencing of all nucleic acids in clinical specimens and alignment against pathogen reference databases, mNGS allows simultaneous detection of bacteria, viruses, fungi, and other pathogens, thereby substantially improving pathogen identification rates (Newborn Science Group of the Pediatrics Branch of Chinese Medical Association et al., 2022; Han and Liu, 2022). Previous studies have demonstrated that mNGS exhibits higher sensitivity than conventional microbiological methods in paediatric bloodstream infections and other severe infections, providing comprehensive aetiological information and demonstrating considerable clinical utility, particularly in complex infections or cases unresponsive to empirical therapy (Wu et al., 2024; Sun et al., 2025).

In neonatal and infant populations, immune immaturity and nonspecific clinical manifestations, together with early-life biological influences, complicate timely infection diagnosis and underscore the need for rapid pathogen detection (Newborn Science Group of the Pediatrics Branch of Chinese Medical Association et al., 2022; Behrami et al., 2026; Sun et al., 2026). mNGS can improve detection rates in suspected infections that are unresponsive to empirical therapy and negative by conventional diagnostics, whilst also enabling identification of fastidious or rare microorganisms (Newborn Science Group of the Pediatrics Branch of Chinese Medical Association et al., 2022; Chen et al., 2022). Despite these advantages, mNGS has limitations including cost, data interpretation complexity, and variable performance across specimen types. Further systematic investigations are needed to delineate its clinical utility and predictive value. In particular, integrating clinical features and laboratory parameters to construct predictive models for mNGS positivity could optimise testing strategies and improve diagnostic efficiency in resource-limited settings; however, large cohort data to support such approaches remain scarce. Systematic reviews have also highlighted that the overall performance and clinical indications of mNGS in paediatric infections require further validation (Gutfreund et al., 2026).

Therefore, we conducted a retrospective analysis of critically ill infants with severe infections admitted to the Children’s Hospital of Soochow University between 1 January 2023 and 31 December 2025, aiming to compare pathogen detection rates of mNGS and conventional culture and to investigate associations between mNGS positivity and clinical laboratory parameters, with the goal of providing evidence-based support and practical decision-making tools for clinical pathogen diagnostics and infection management.

## 2 Methods

### 2.1 Study population and grouping

This single-centre retrospective study included critically ill infants younger than 1 year of age with severe infections admitted to the Children’s Hospital of Soochow University between 1 January 2023 and 31 December 2025, who underwent both mNGS and conventional culture testing. The diagnosis of severe infection was based on the relevant criteria outlined in *Practical Pediatrics* (Zhao, 2011). Infants with confirmed genetic metabolic disorders, chromosomal abnormalities, or immunodeficiencies were excluded.

The study protocol was approved by the Ethics Committee of the Children’s Hospital of Soochow University (approval no. 2025CS239) and conducted in accordance with the Declaration of Helsinki and its subsequent amendments. Written informed consent was obtained from the guardians of all enrolled infants, including consent for the use and disclosure of clinical data.

For comparison of positivity rates between mNGS and conventional culture, analyses were performed on a per-specimen basis. All infants were classified into mNGS-positive and mNGS-negative groups according to mNGS results, and clinical characteristics and laboratory parameters were compared between groups on a per-patient basis.

### 2.2 Clinical data and laboratory assessment

Clinical data were retrospectively collected from the electronic medical record system, including demographic characteristics (sex, age, gestational age, and birth weight) and infection-related information (infection site and type). Conventional microbiological culture and mNGS results were obtained from blood, sputum, cerebrospinal fluid, and other body fluid specimens. Laboratory parameters closest to the time of mNGS testing were recorded, encompassing complete blood count (white blood cell count, red blood cell count, haemoglobin, platelet count, and differential leucocyte counts), inflammatory markers (CRP and PCT), and biochemical indices including cholinesterase and electrolytes. mNGS and culture results were interpreted by clinicians in conjunction with each patient’s clinical presentation, imaging findings, and laboratory data to determine the clinical relevance of detected pathogens. All identified pathogens were classified and analysed by type, including bacteria, viruses, and fungi.

Since the implementation of rapid mNGS in our institution, mNGS has been routinely applied in parallel with conventional microbiological testing for suspected severe infections. For suspected bloodstream infection, blood culture and plasma cfDNA mNGS samples were collected on the same day. When simultaneous collection was not feasible, specimens were obtained within the same infectious episode (within 24 h) and before major antimicrobial adjustment whenever possible. For other specimen types, mNGS and culture samples were collected under the same principle of minimal temporal separation.

### 2.3 mNGS laboratory procedure and positivity criteria

For blood specimens, approximately 3–5 mL of peripheral whole blood was collected in EDTA or plasma preparation tubes and centrifuged for plasma separation prior to cfDNA-based mNGS analysis. Plasma cfDNA was extracted from approximately 1–2 mL of plasma using the QIAamp Circulating Nucleic Acid Kit (Qiagen, Hilden, Germany) according to the manufacturer’s protocol. Purified nucleic acids were eluted in 50 μL of elution buffer, and cfDNA concentration and fragment-size distribution were assessed using the Agilent 2,100 Bioanalyzer prior to library preparation.

DNA libraries were constructed using the NEBNext Ultra II FS DNA Library Prep Kit for Illumina (New England Biolabs, Ipswich, MA, USA), including end repair/dA-tailing, adapter ligation, and PCR amplification. RNA sequencing was performed only in cases with predefined clinical suspicion of RNA viral infection. For these cases, viral RNA was extracted from a parallel aliquot of the same original clinical specimen using the QIAamp Viral RNA Mini Kit (Qiagen, Hilden, Germany), in parallel with DNA/cfDNA extraction. The extracted RNA was then subjected to reverse transcription and RNA library preparation using the NEBNext Ultra II Directional RNA Library Prep Kit for Illumina (New England Biolabs, Ipswich, MA, USA), according to the manufacturer’s protocol. Because RNA-based sequencing was performed selectively as part of routine clinical testing, RNA-specific concentration values were not systematically recorded in the retrospective clinical reporting system and therefore could not be reliably retrieved on a per-sample basis. All libraries passing quality control, with an input nucleic acid amount of ≥25 ng, were sequenced on an Illumina NextSeq 550Dx platform, generating approximately 20 million single-end 75 bp reads per sample. Sequencing reads were quality filtered, trimmed, and aligned to the human reference genome (hs37d5) using Bowtie2 for host depletion. Remaining reads were taxonomically classified using Kraken2 (v2.0.7) against a curated NCBI GenBank microbial database.

Pathogen identification applied a dual-threshold strategy, requiring ≥1 species-specific read for *Mycobacterium tuberculosis* complex, Nocardia spp., and *Legionella pneumophila*, and ≥3 non-overlapping reads for all other organisms, based on internal negative-control distributions to reduce stochastic and alignment artefacts in low-biomass plasma samples. To control contamination, all taxa were compared against batch-specific negative template controls (NTCs), with reads per million (RPM) calculated and a normalised enrichment ratio (RPMr = RPM_sample/RPM_NTC) applied at the taxon level; when RPM_NTC was zero, a pseudo-count of 1 was used. Taxa with RPMr <10 were excluded prior to interpretation. Final pathogen assignment integrated sequencing results with clinical presentation, laboratory findings, imaging, and conventional microbiological testing, and organisms were classified by multidisciplinary review as confirmed, probable, or unlikely pathogens.

### 2.4 Statistical analysis

All statistical analyses were performed using SPSS version 27.0 (IBM Corp., Armonk, NY, USA) and R version 4.1.2 (R Foundation for Statistical Computing, Vienna, Austria). Continuous variables were tested for normality and presented as mean ± SD or median (IQR) as appropriate, with group comparisons performed using the independent-samples t-test or Mann–Whitney U test. Categorical variables were expressed as counts (percentages) and compared using the χ^2^ test or Fisher’s exact test. Because mNGS and conventional culture results were obtained from the same specimens, positivity rates were compared using McNemar’s test for paired proportions.

Univariable analysis was used to identify candidate variables associated with mNGS positivity, which were then entered into a LASSO regression model with 10-fold cross-validation for variable selection based on non-zero coefficients. Selected variables were subsequently included in a multivariable logistic regression model to assess independent associations. Model significance was evaluated using the likelihood ratio test, and calibration was assessed with the Hosmer–Lemeshow test.

Predictive performance of individual predictors and the combined model was evaluated using ROC curve analysis, with AUCs and 95% CIs reported. Optimal cut-off values were determined by the Youden index, with corresponding sensitivity and specificity. Internal validation was performed using 10-fold cross-validation of the full modelling pipeline, with bootstrap resampling (2,000 iterations) used to estimate the 95% CI of the cross-validated AUC. Given the retrospective consecutive design, no formal sample size calculation was performed. Sample adequacy was supported by an EPV of 18.7 (56 mNGS-positive cases, 3 predictors), exceeding the recommended threshold of 10, and post-hoc power exceeded 99% for ROC analysis at the observed AUC of 0.842 (*α* = 0.05). All tests were two-sided, and *p* < 0.05 was considered statistically significant.

## 3 Results

### 3.1 Demographic and clinical characteristics

**Figure 1:**
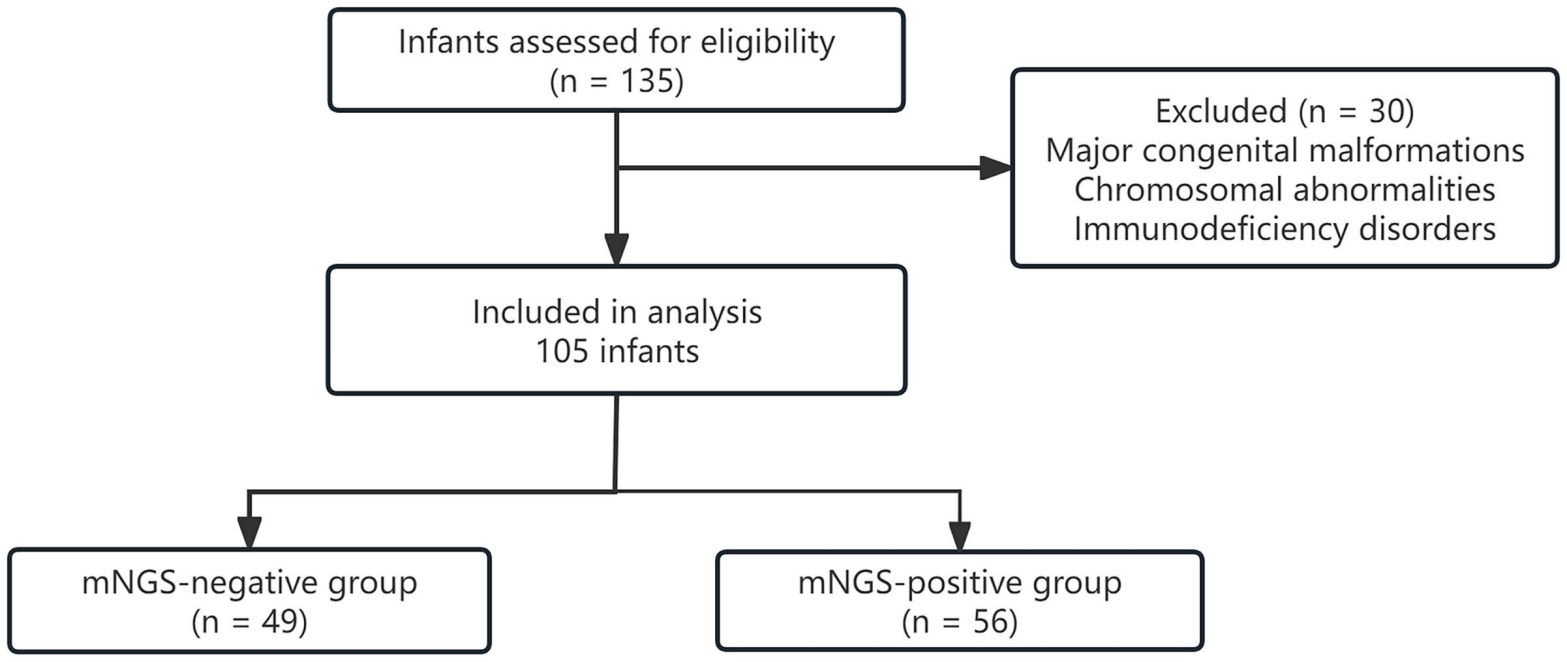
Flowchart of the study. mNGS, metagenomic next-generation sequencing.

A detailed flowchart of the study enrollment process is presented in Figure 1. A total of 135 infants with severe infections were initially identified. Thirty were excluded due to major congenital malformations, chromosomal abnormalities, or immunodeficiency disorders, leaving 105 infants and 153 biological specimens for analysis. Demographic and baseline characteristics are summarised in Table 1. Of the 105 infants, 49 were classified into the mNGS-negative group and 56 into the mNGS-positive group. There were 59 males (56.2%) and 46 females (43.8%). Sixty-four infants (60.9%) were in the neonatal period (0–28 days) and 41 (39.1%) in the infant period (29 days to 12 months). With respect to infection site, severe pneumonia was the most common, affecting 76 infants (72.4%), followed by sepsis (including septicaemia) in 31 (29.5%), central nervous system infections in 22 (21.0%), intra-abdominal infections in 11 (10.5%), gastrointestinal infections in 8 (7.6%), and urinary tract infections in 7 (6.7%). Regarding infection type, 82 cases (78.1%) were community-acquired and 23 (21.9%) were hospital-acquired.

**Table 1:** Demographic and clinical characteristics of critically ill infants (*n* = 105).

Characteristic	Category	*n*	%
Sex	Male	59	56.2
	Female	46	43.8
Age group	Neonatal (0–28 days)	64	60.9
	Infant (29 days–12 months)	41	39.1
Infection site	Severe pneumonia	76	72.4
	Septicaemia	31	29.5
	Central nervous system infection	22	21.0
	Gastrointestinal infection	8	7.6
	Urinary tract infection	7	6.7
	Intra-abdominal infection	11	10.5
Infection type	Community-acquired	82	78.1
	Hospital-acquired	23	21.9

Individual patients may have had more than one concurrent infection site, and percentages therefore sum to more than 100%.

### 3.2 Comparison of mNGS and conventional culture positivity

The positivity rates of mNGS and conventional microbiological culture across different specimen types are summarised in Table 2. Overall, mNGS demonstrated a significantly higher positivity rate than conventional culture (all specimens: 44.4%, 68/153 vs. 21.6%, 33/153; *p* = 0.001). Stratified analysis by specimen type showed that in blood specimens, the positivity rate of mNGS was 34.3% (24/70), higher than that of conventional culture (14.3%, 10/70). In sputum specimens, mNGS positivity was 68.8% (33/48), significantly higher than conventional culture (37.5%, 18/48). For other specimen types, mNGS detected pathogens in 6 cases, whereas conventional culture was positive in only 1 case (all *p* < 0.05). In cerebrospinal fluid specimens, the positivity rates were comparable between mNGS and conventional culture (17.2%, 5/29 vs. 13.8%, 4/29), with no statistically significant difference.

**Table 2:** Comparison of positivity rates between mNGS and conventional culture.

Specimen type	mNGS positive	mNGS negative	Conventional culture positive	Conventional culture negative	*p* value
All specimens	68	85	33	120	0.001
Blood	24	46	10	60	0.006
Sputum	33	15	18	30	<0.001
Cerebrospinal fluid	5	24	4	25	0.869
Other	6	0	1	5	0.045

mNGS, metagenomic next-generation sequencing.

### 3.3 Pathogen detection

The pathogen detection results are summarised in Table 3. mNGS identified a total of 132 pathogens, including 106 bacteria (80.3%), 23 viruses (17.4%), and 3 fungi (2.3%). In contrast, conventional microbiological culture detected 23 pathogens, comprising 22 bacteria (95.7%) and 1 fungus (4.3%). Amongst bacteria detected by mNGS, the most frequently identified species were *Staphylococcus epidermidis* and *Streptococcus pneumoniae* (15 cases each, 14.2%), *Enterococcus faecium* (13 cases, 12.3%), *Escherichia coli* (9 cases, 8.5%), and *Staphylococcus aureus* (8 cases, 7.5%). By comparison, conventional culture primarily detected *S. aureus* (5 cases, 22.7%), *E. coli* (5 cases, 22.7%), *Pseudomonas aeruginosa* (3 cases, 13.6%), and *E. faecium* (2 cases, 9.1%). mNGS also identified several pathogens that are difficult to detect by conventional culture, including the *Mycobacterium tuberculosis* complex, *Gardnerella vaginalis*, *Ureaplasma parvum*, *Ureaplasma urealyticum*, and *Mycoplasma pneumoniae*. Amongst viruses, mNGS detected five viral species in 23 cases, with human cytomegalovirus (10 cases, 43.5%) and human herpesvirus (10 cases, 43.5%) being the most prevalent. The total number of culture-positive detections in Table 3 (23 case-level species detections) is lower than the number of culture-positive specimens in Table 2 and Sauberan et al. (2026), because multiple specimens from the same patient yielding the same organism were counted once at the case-level species-detection level in Table 3 but as separate specimens in Table 2.

**Table 3:** Pathogen detection by mNGS and conventional culture.

Pathogen type	Pathogen	mNGS detection	Conventional culture detection	Detected by both
Bacteria	*Staphylococcus epidermidis*	15	3	3
	*Streptococcus pneumoniae*	15	0	0
	*Enterococcus faecium*	13	2	1
	*Escherichia coli*	9	5	2
	*Staphylococcus aureus*	8	5	4
	*Acinetobacter baumannii*	7	0	0
	*Lactococcus lactis*	6	0	0
	*Enterococcus faecalis*	4	0	0
	*Pseudomonas aeruginosa*	4	3	1
	*Klebsiella aerogenes*	4	0	0
	*Staphylococcus hominis*	4	0	0
	*Ureaplasma parvum*	3	0	0
	*Bacillus cereus*	3	0	0
	*Citrobacter freundii*	2	0	0
	*Staphylococcus capitis*	2	0	0
	*Klebsiella variicola*	2	0	0
	*Mycobacterium tuberculosis* complex	1	0	0
	*Gardnerella vaginalis*	1	0	0
	*Mycoplasma pneumoniae*	1	0	0
	*Ureaplasma urealyticum*	1	0	0
	*Staphylococcus cohnii*	1	0	0
	*Klebsiella pneumoniae*	0	2	0
	*Acinetobacter johnsonii*	0	1	0
	*Haemophilus influenzae*	0	1	0
Subtotal (Bacteria)		106	22	11
Viruses	Human cytomegalovirus	10	0	0
	Human herpesvirus	10	0	0
	Human respiratory syncytial virus	1	0	0
	Norovirus	1	0	0
	Human parainfluenza virus	1	0	0
Subtotal (Viruses)		23	0	0
Fungi	*Aspergillus flavus*	2	0	0
	*Candida albicans*	1	1	1
Subtotal (Fungi)		3	1	1

mNGS, metagenomic next-generation sequencing. Detection counts are reported as case-level species detections; repeated detection of the same organism from multiple specimens of the same patient was counted once. A single specimen could yield multiple species. The “Detected by Both” column reflects case-level species co-detection, whereas specimen-level concordance is reported in Section 3.4.

### 3.4 Clinical impact of discordant mNGS results

Of the 68 mNGS-positive specimens, 27 were concurrently positive by conventional culture, whilst 41 were mNGS-positive but culture-negative. Conversely, 6 of 33 culture-positive specimens (18.2%) were mNGS-negative. These six culture-positive, mNGS-negative specimens yielded seven culture isolates, including *Escherichia coli* (*n* = 3), *Klebsiella pneumoniae* (*n* = 2), *Acinetobacter johnsonii* (*n* = 1), and *Haemophilus influenzae* (*n* = 1). Amongst pathogens identified by mNGS, those belonging to species with zero conventional culture recovery in this cohort totalled 82 detections and fell into three categories. Conventional bacteria not recoverable by culture at any instance comprised the largest group (*n* = 50), with *Streptococcus pneumoniae* (*n* = 15), *Acinetobacter baumannii* (*n* = 7), and *Lactococcus lactis* (*n* = 6) being the most frequent. Viruses, which are inherently undetectable by culture, accounted for 23 detections, including human cytomegalovirus (*n* = 10), human herpesvirus (*n* = 10), human respiratory syncytial virus (*n* = 1), norovirus (*n* = 1), and human parainfluenza virus (*n* = 1). Atypical and fastidious bacteria not amenable to routine culture included *Ureaplasma parvum* (*n* = 3), *Mycoplasma pneumoniae* (*n* = 1), *Ureaplasma urealyticum* (*n* = 1), *Gardnerella vaginalis* (*n* = 1), and *Mycobacterium tuberculosis* complex (*n* = 1). Additionally, *Aspergillus flavus* (*n* = 2) was detected exclusively by mNGS. An additional 38 mNGS detections belonged to species also recoverable by culture (e.g., *S. epidermidis*, *E. faecium*), representing cases where mNGS provided additional case-level detection beyond culture within species also amenable to conventional recovery. Based on review of clinical records, mNGS findings with no corresponding culture recovery resulted in antimicrobial management changes in 17 patients, comprising antibiotic regimen modification in 12 and initiation of antiviral therapy in 5 with cytomegalovirus or herpesvirus detection.

### 3.5 Comparison of laboratory parameters between mNGS-positive and mNGS-negative patients

Laboratory findings for the mNGS-negative and mNGS-positive groups are summarised in Table 4 and Supplementary Table S1. No significant differences were observed between the two groups in haematologic parameters, including white blood cell count, red blood cell count, haemoglobin, platelet count, and most leukocyte differentials (all *p* > 0.05). However, eosinophil-related indices were significantly lower in the mNGS-positive group, including both eosinophil percentage and absolute eosinophil count. Red blood cell parameters were also reduced, particularly mean corpuscular volume (MCV) and MCH (both *p* < 0.05). Inflammatory markers were elevated in the mNGS-positive group, with significantly elevated CRP and PCT levels (both *p* < 0.05). Amongst biochemical parameters, cholinesterase and serum calcium levels were significantly lower in the mNGS-positive group (*p* < 0.05), whereas other liver, renal, and metabolic indices showed no statistically significant differences (all *p* > 0.05).

**Table 4:** Comparison of laboratory parameters between mNGS-positive and mNGS-negative patients.

Variable	mNGS negative (*n* = 49)	mNGS positive (*n* = 56)	*p* value
Complete blood count and inflammatory markers			
White blood cell count (×10^9^/L)	10.71 (6.68, 15.11)	11.80 (6.89, 18.45)	0.537
Haemoglobin (g/L)	116.00 (100.00, 145.00)	108.50 (92.75, 123.50)	0.060
Platelet count (×10^9^/L)	206.00 (154.00, 385.00)	179.50 (88.25, 336.25)	0.082
Neutrophil percentage (%)	57.60 (40.60, 66.70)	65.40 (40.70, 73.50)	0.261
Eosinophil percentage (%)	1.60 (0.50, 3.00)	0.60 (0.20, 2.42)	0.020
Basophil percentage (%)	0.20 (0.10, 0.30)	0.10 (0.10, 0.30)	0.059
Lymphocyte percentage (%)	30.10 (19.40, 39.00)	23.95 (15.83, 44.02)	0.377
Monocyte percentage (%)	10.10 (6.70, 14.90)	9.85 (6.55, 14.10)	0.736
Mean corpuscular volume (MCV)(fL)	99.00 (89.00, 107.00)	90.00 (87.00, 99.00)	0.002
Mean corpuscular haemoglobin (pg)	33.51 ± 3.77	30.76 ± 3.54	<0.001
Absolute eosinophil count (×10^9^/L)	0.16 (0.04, 0.31)	0.08 (0.02, 0.18)	0.039
C-reactive protein (CRP) (mg/L)	18.73 (2.24, 53.00)	36.44 (4.68, 107.62)	0.042
Procalcitonin (PCT) (ng/mL)	0.36 (0.12, 2.90)	1.78 (0.54, 8.36)	0.005
Biochemistry			
Total protein (g/L)	50.10 (41.80, 56.00)	49.30 (42.03, 56.75)	1.000
Albumin (g/L)	34.00 (29.30, 37.40)	33.25 (28.50, 36.20)	0.400
Globulin (g/L)	16.20 (12.20, 19.30)	15.30 (11.50, 21.32)	0.865
Aspartate aminotransferase (AST) (U/L)	38.50 (24.30, 55.80)	41.65 (27.90, 69.22)	0.196
Alanine aminotransferase (ALT) (U/L)	14.90 (7.70, 28.90)	18.80 (9.50, 49.50)	0.079
Lactate dehydrogenase (LDH) (U/L)	361.80 (289.00, 649.10)	405.90 (317.38, 588.27)	0.360
Cholinesterase (U/L)	5713.84 ± 2109.01	4652.46 ± 1932.97	0.009
Calcium (mmol/L)	2.33 (2.23, 2.46)	2.27 (2.10, 2.35)	0.017

### 3.6 Variable selection and regression analysis

**Figure 2:**
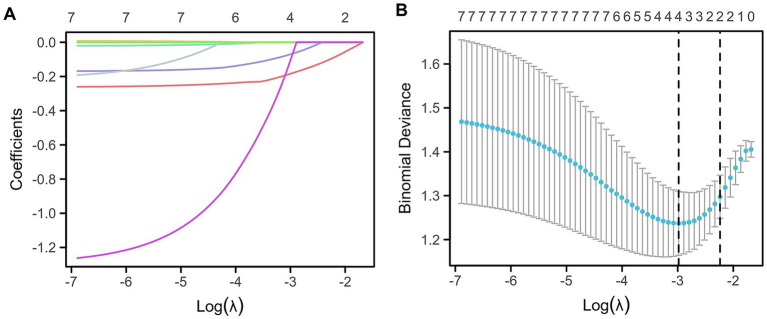
LASSO regression variable selection process. **(A)** Regression coefficient trajectories of candidate variables in the LASSO model. **(B)** Ten-fold cross-validation for determining the optimal penalty parameter (*λ*). LASSO, least absolute shrinkage and selection operator.

Eight variables with statistical significance were identified through univariable analysis and subsequently included in a LASSO regression model (Table 5; Figure 2). LASSO regression selected five variables with non-zero coefficients: eosinophil percentage, MCH, PCT, cholinesterase, and serum calcium. These five variables were further entered into a multivariable logistic regression model. The analysis revealed that MCH, cholinesterase, and PCT were independently associated with mNGS positivity. Higher MCH (OR = 0.755, 95%CI: 0.657–0.869, *p* < 0.001) and cholinesterase levels (OR = 0.999, 95%CI: 0.999–1.000, *p* = 0.001) were associated with a decreased risk of mNGS positivity, whereas elevated PCT levels (OR = 1.180, 95%CI: 1.050–1.320, *p* = 0.006) were associated with an increased likelihood of mNGS detection.

**Table 5:** LASSO selection and logistic regression analysis of variables associated with mNGS positivity.

Variable	Univariate *p* value	Selected by LASSO	Coefficient (LASSO)	Adjusted OR (95% CI)	*p* value
Eosinophil percentage	0.020	Yes	−0.215	0.806 (0.640–1.016)	0.067
Absolute eosinophil count	0.039	No	—	—	—
MCV	0.002	No	—	—	—
MCH	<0.001	Yes	−0.281	0.755 (0.657–0.869)	<0.001
CRP	0.042	No	—	—	—
PCT	0.005	Yes	0.148	1.180 (1.050–1.320)	0.006
Cholinesterase	0.009	Yes	−0.0004	0.999 (0.999–1.000)	0.001
Calcium	0.017	Yes	−0.544	0.580 (0.296–1.138)	0.115

mNGS, metagenomic next-generation sequencing; MCV, mean corpuscular volume; MCH, mean corpuscular haemoglobin; CRP, C-reactive protein; PCT, procalcitonin.

### 3.7 ROC curve analysis

**Figure 3:**
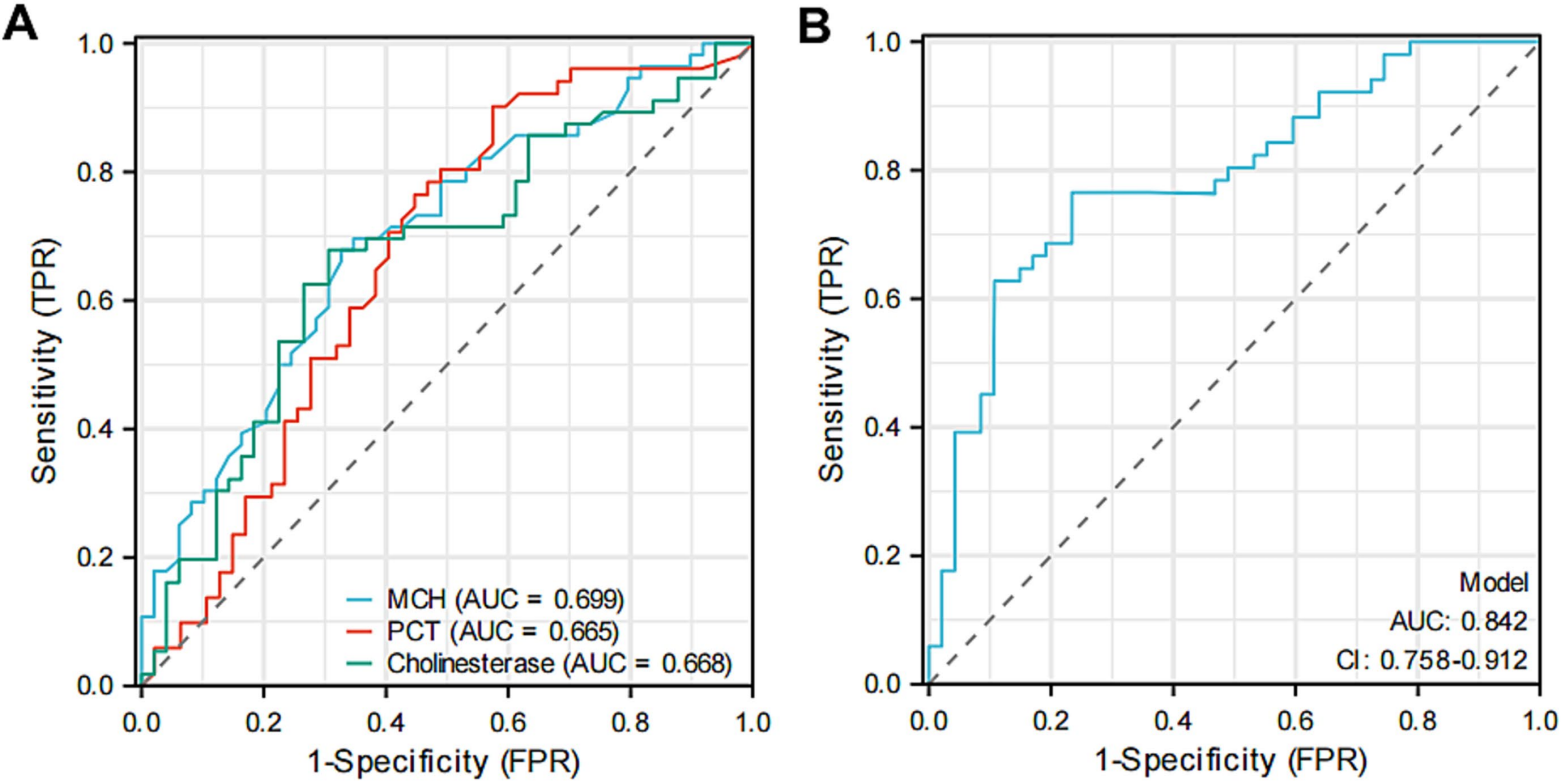
ROC curves of single indicators and combined model. **(A)** ROC curves of individual indicators, showing the predictive performance of MCH, cholinesterase, and PCT for mNGS positivity. **(B)** ROC curve of the combined model. The model integrating MCH, cholinesterase, and PCT demonstrates a higher area under the curve (AUC), indicating superior discriminative ability compared with any single indicator. mNGS, metagenomic next-generation sequencing; MCH, mean corpuscular haemoglobin; PCT, procalcitonin.

ROC curve analysis was performed for each predictor identified through LASSO and multivariable logistic regression, alongside the combined model (Table 6; Figure 3). Amongst individual predictors, MCH achieved the highest discriminatory performance (AUC = 0.699, 95%CI: 0.592–0.797; sensitivity 67.9%, specificity 67.3% at a cut-off ≤31.7 pg), followed by cholinesterase (AUC = 0.668, 95%CI: 0.565–0.763; sensitivity 67.9%, specificity 69.4% at cut-off ≤4,868 U/L) and PCT (AUC = 0.665, 95%CI: 0.548–0.769; sensitivity 90.2%, specificity 42.6% at cut-off ≥0.210 ng/mL). The combined model incorporating all three variables yielded an AUC of 0.842 (95%CI: 0.758–0.912), with sensitivity 87.2% and specificity 81.6% at an optimal cut-off of 0.552. Overall model significance was confirmed by the likelihood ratio test (χ^2^ = 25.59, *p* < 0.001), and goodness-of-fit was supported by the Hosmer–Lemeshow test (χ^2^ = 9.31, *p* = 0.317).

**Table 6:** ROC analysis of key predictors for mNGS positivity.

Variable/Model	AUC (95% CI)	Optimal cutoff	Sensitivity	Specificity
MCH	0.699 (0.592–0.797)	≤31.70	67.9%	67.3%
Cholinesterase	0.668 (0.565–0.763)	≤4868.00	67.9%	69.4%
PCT	0.665 (0.548–0.769)	≥0.210	90.2%	42.6%
MCH + Cholinesterase + PCT	0.842 (0.758–0.912)	0.552	87.2%	81.6%

mNGS, metagenomic next-generation sequencing; MCH, mean corpuscular haemoglobin; PCT, procalcitonin.

To evaluate model stability and correct for optimism, 10-fold cross-validation was applied to the complete modelling pipeline, with LASSO variable selection and logistic regression fitting performed independently within each training fold. The cross-validated AUC was 0.817 (95%CI: 0.696–0.899), compared with the apparent AUC of 0.842, yielding an optimism estimate of 0.025. Across the 10-folds, MCH and cholinesterase were selected in all folds (10/10), PCT and serum calcium each in 8 of 10 folds, demonstrating robust variable selection stability for the three identified predictors (Supplementary Table S2).

## 4 Discussion

In this study, we compared pathogen detection rates between mNGS and conventional culture in critically ill infants and analysed associations between mNGS positivity, clinical characteristics, and laboratory parameters. Using LASSO regression followed by multivariable logistic regression, we developed a predictive model for mNGS positivity. Our results demonstrated that mNGS significantly outperformed conventional culture in pathogen detection, particularly for fastidious organisms and mixed infections. Furthermore, mNGS positivity was independently associated with MCH, cholinesterase, and PCT, underscoring the potential utility of these parameters in identifying candidates for mNGS testing amongst critically ill infants.

The overall mNGS positivity rate was significantly higher than that of conventional culture in the present cohort, consistent with previous studies reporting improved sensitivity of cfDNA-based sequencing approaches across a range of paediatric infectious disease settings, including immunocompromised and critically ill patients (Zhang et al., 2023; Elbehiry and Abalkhail, 2025; Balks et al., 2025; Adolph et al., 2026; Armstrong et al., 2019). Conventional culture depends on viable pathogen growth and isolation, which limits its ability to detect anaerobic bacteria, viruses, and fungi. In contrast, mNGS enables culture-independent, simultaneous detection of a broad spectrum of pathogens through whole-genome sequencing, making it particularly advantageous for fastidious or difficult-to-culture organisms (Sun et al., 2025; Wei et al., 2024). Stratified analysis by specimen type showed that mNGS had significantly higher positivity rates than culture in blood and sputum samples, indicating improved sensitivity for bloodstream and respiratory infections, particularly in the context of polymicrobial infections. These findings are consistent with previous reports and further support the clinical value of mNGS in complex or microbiologically unexplained infections (Chen et al., 2024; Qian et al., 2023). Cases with positive culture but negative mNGS may be attributed to low pathogen burden below the cfDNA detection threshold, high background of host-derived cfDNA, localised infection with limited nucleic acid release into circulation, heterogeneous pathogen distribution in mixed infections, or pre-analytical factors affecting nucleic acid recovery. Therefore, a negative mNGS result should not be used to exclude infection when microbiological and clinical evidence is supportive. No significant difference between mNGS and culture was observed in CSF specimens, likely reflecting the intrinsic limitations of CSF for mNGS, including low pathogen load, limited sample volume, and a high proportion of host-derived nucleic acids that may compromise pathogen signal detection (Miller et al., 2019; Feng et al., 2026; Wilson et al., 2019). Given that only five CSF samples were mNGS-positive in this cohort, conclusions regarding performance in CSF require further validation in larger, dedicated studies. Notably, in a considerable proportion of discordant cases, mNGS-specific findings directly informed clinical management, further underscoring its added diagnostic and therapeutic value beyond conventional microbiological methods.

In this study, LASSO regression analysis identified clinical indicators significantly associated with mNGS positivity, particularly MCH, cholinesterase, and PCT. At the immunological level, the core feature of severe infections and sepsis is a dysregulated host immune response, characterised by excessive inflammation causing tissue damage and simultaneous immunosuppression increasing the risk of pathogen reactivation (Zhang et al., 2024; Cajander et al., 2024). This dysregulated immune state is accompanied by dynamic changes in circulating cell-free DNA, which are influenced by both increased cellular release and altered nuclease-mediated clearance (Lenz et al., 2022). This complex immune status is closely linked to alterations in MCH, cholinergic regulation, and amplification of inflammatory cascades (You, 2025), providing a coherent physiological explanation for their predictive value in mNGS positivity.

Amongst critically ill infants, low MCH likely reflects pre-existing nutritional iron deficiency, as erythrocyte indices change slowly relative to the time course of acute infection. Iron deficiency impairs multiple components of innate immunity, including neutrophil bactericidal capacity, oxidative burst activity (Hassan et al., 2016; Walter et al., 1986), and natural killer cell cytotoxicity against virus-infected targets (Schimmer et al., 2025), potentially increasing susceptibility to pathogens less recoverable by conventional culture. This is consistent with the predominance of viruses and atypical bacteria amongst mNGS-exclusive findings in the present cohort, and may partly explain the independent association of MCH with mNGS positivity. Whether this reflects a direct mechanistic pathway or a broader indicator of immune vulnerability warrants prospective evaluation. Cholinesterase participates in the cholinergic anti-inflammatory pathway, whereby vagal acetylcholine suppresses pro-inflammatory cytokine release from immune cells (Chen et al., 2026). In severe infections, reduced cholinesterase activity signals cholinergic dysregulation and systemic immune exhaustion (Neu et al., 2024), conditions associated with impaired host defences and vulnerability to a broader spectrum of pathogens, including those not recoverable by conventional culture. The independent association of cholinesterase with mNGS positivity in this cohort aligns with its established role as a marker of immune dysfunction severity in critical illness. PCT is a well-validated marker of bacterial infection, rising markedly in the early phase of systemic infection and correlating with pathogen burden and disease severity (Meisner, 2014). Its association with mNGS positivity in this cohort is consistent with prior evidence that higher PCT levels reflect a greater likelihood of true infection requiring pathogen identification, rather than non-infectious inflammation (Simon et al., 2004). Taken together, MCH, cholinesterase, and PCT capture complementary dimensions of the host response to infection, nutritional immune status, autonomic immunomodulation, and acute-phase bacterial signalling, providing a biologically coherent basis for their combined predictive value for mNGS positivity.

Despite the diagnostic advantages demonstrated in this study, several limitations merit acknowledgement. First, the single-centre retrospective design and restricted sample size may introduce selection bias and limit generalisability. Second, high cost and dependence on specialised bioinformatics infrastructure remain barriers to the widespread adoption of mNGS, particularly in resource-limited settings. Third, sensitivity may be compromised by prior antibiotic exposure, which may reduce detectable microbial nucleic acids, and by the predominance of host-derived DNA in blood and body fluid specimens. Because this retrospective study was based on routine clinical mNGS reports, complete run-level sequencing metrics, including host-derived read proportion, total microbial read burden, and host-to-microbial read ratios, were not systematically available in the study-accessible database. Therefore, correlations between microbial read burden and cfDNA concentration or inflammatory biomarkers such as PCT and CRP could not be reliably assessed. Future prospective studies should retain these sequencing quality metrics to better define the relationship between host background, microbial read burden, and mNGS positivity. Conversely, the inherent high sensitivity of mNGS risks detecting colonising rather than causally relevant organisms, particularly in neonatal populations where background microbial cell-free DNA may be present even in clinically well-appearing infants (Sauberan et al., 2026). Future work should focus on standardising clinical indications, retaining sequencing quality metrics in prospective datasets, refining host-depletion protocols, and prospectively validating integrated diagnostic models incorporating mNGS alongside conventional methods.

## 5 Conclusion

This study demonstrates that mNGS provides significantly superior pathogen detection compared with conventional culture in critically ill infants, particularly for fastidious pathogens and mixed infections. MCH, cholinesterase, and PCT were identified as independent predictors of mNGS positivity, and a combined model incorporating these three parameters substantially improved predictive performance, offering more targeted guidance for clinical decision-making. In clinical practise, mNGS should be considered when empirical antimicrobial therapy fails or conventional diagnostics yield negative results, facilitating pathogen identification and guiding targeted therapy. With further technological optimisation and cost reduction, mNGS holds considerable potential for broader application in the early diagnosis and personalised management of severe infections in infants.

## Data Availability

The raw sequencing data are not publicly available due to privacy and regulatory restrictions. Access to the data is controlled and may be granted upon reasonable request to the corresponding author, subject to ethical and regulatory approvals.
