# Supplementary material for: Clinical utility of metagenomic next-generation sequencing in infants with severe infections
Source: Front Microbiol. 2026 Jul 16;17:1842600. doi: 10.3389/fmicb.2026.1842600 (PMC13422504; doi:10.3389/fmicb.2026.1842600)
Supplement: Supplementary file 1 [file Table_1.DOCX]

**Table S1. Comparison of Laboratory Parameters Between mNGS-Positive and mNGS-Negative Patients**

| **Variable** | **NGS negative**  **(n=49)** | **NGS positive**  **(n=56)** | ***P* value** |
| --- | --- | --- | --- |
| **Complete blood count and inflammatory markers** | | | |
| Red blood cell count (×10^12^/L) | 3.58 (3.02, 4.09) | 3.54 (3.15, 3.97) | 0.865 |
| Hematocrit (%) | 0.34 (0.29, 0.44) | 0.33 (0.28, 0.37) | 0.093 |
| Mean corpuscular hemoglobin concentration (MCHC)(g/L) | 336.00 (328.00, 346.00) | 334.50 (323.75, 341.00) | 0.262 |
| Red cell distribution width (RDW)(%) | 15.20 (14.30, 16.50) | 15.75 (14.80, 17.62) | 0.103 |
| Absolute neutrophil count (×10⁹/L) | 4.84 (3.24, 9.94) | 7.20 (2.49, 12.48) | 0.273 |
| Absolute basophil count (×10⁹/L) | 0.02 (0.01, 0.03) | 0.01 (0.01, 0.03) | 0.068 |
| Absolute lymphocyte count (×10⁹/L) | 3.13 (1.91, 4.56) | 2.83 (1.52, 3.77) | 0.214 |
| Absolute monocyte count (×10⁹/L) | 0.89 (0.54, 1.61) | 1.04 (0.65, 1.95) | 0.683 |
| Plateletcrit (%) | 0.24 (0.18, 0.35) | 0.21 (0.12, 0.37) | 0.145 |
| Mean platelet volume (MPV)(fL) | 10.49 ± 1.57 | 10.66 ± 1.22 | 0.556 |
| Platelet distribution width (PDW) (%) | 14.80 (11.05, 16.60) | 16.10 (12.30, 16.70) | 0.163 |
| **Biochemistry** | | | |
| Total bilirubin (μmol/L) | 50.20 (16.70, 100.10) | 38.95 (12.65, 73.75) | 0.329 |
| Direct bilirubin (μmol/L) | 10.60 (3.70, 13.70) | 9.95 (4.50, 15.43) | 0.845 |
| Indirect bilirubin (μmol/L) | 28.90 (7.30, 88.00) | 17.15 (4.95, 53.42) | 0.199 |
| Gamma-glutamyl transferase (GGT) (U/L) | 78.80 (52.80, 119.40) | 92.50 (56.08, 190.07) | 0.406 |
| Alkaline phosphatase (ALP) (U/L) | 231.55 (171.70, 274.80) | 222.00 (151.10, 343.23) | 0.711 |
| Total bile acids (μmol/L) | 7.88 (2.42, 16.86) | 3.51 (1.43, 10.31) | 0.103 |
| Blood urea nitrogen (BUN) (mmol/L) | 3.96 (2.75, 7.92) | 5.38 (2.69, 17.95) | 0.371 |
| Creatinine (μmol/L) | 25.00 (16.20, 43.40) | 23.60 (10.46, 39.85) | 0.562 |
| Uric acid (μmol/L) | 170.20 (126.30, 272.80) | 182.20 (124.20, 259.50) | 0.945 |
| α-Hydroxybutyrate dehydrogenase (α-HBDH) (U/L) | 297.00 (203.50, 526.75) | 322.00 (235.00, 456.35) | 0.558 |
| Creatine kinase (CK) (U/L) | 111.25 (76.10, 245.45) | 106.80 (54.15, 218.20) | 0.670 |
| Magnesium (mmol/L) | 0.94 (0.88, 1.00) | 0.92 (0.86, 1.07) | 0.965 |
| Total cholesterol (mmol/L) | 2.90 ± 1.00 | 2.60 ± 1.11 | 0.169 |
| Triglycerides (mmol/L) | 0.86 (0.59, 1.19) | 0.94 (0.54, 1.41) | 0.472 |
| Prealbumin (mg/L) | 102.00 (73.00, 118.75) | 97.50 (68.25, 119.75) | 0.565 |
| Lipase (U/L) | 10.70 (8.90, 13.88) | 11.70 (8.95, 14.18) | 0.560 |
| Albumin/globulin ratio (A/G) | 2.18 (1.87, 2.60) | 2.15 (1.50, 2.72) | 0.684 |

Note: mNGS, metagenomic next-generation sequencing
