# Supplementary material for: Clinical utility of metagenomic next-generation sequencing in infants with severe infections
Source: Front Microbiol. 2026 Jul 16;17:1842600. doi: 10.3389/fmicb.2026.1842600 (PMC13422504; doi:10.3389/fmicb.2026.1842600)
Supplement: Supplementary file 2 [file Table_2.DOCX]

**Supplementary Table2. Variable Selection Stability and Fold-level AUC Across Ten-fold Cross-validation**

| **Fold** | **Eosinophil percentage** | **MCV** | **MCH** | **Absolute eosinophil count** | **CRP** | **PCT** | **Cholinesterase** | **Serum calcium** | **Fold AUC** |
| --- | --- | --- | --- | --- | --- | --- | --- | --- | --- |
| Fold 1 | – | **√** | **√** | – | – | – | **√** | – | 0.400 |
| Fold 2 | – | – | **√** | – | – | **√** | **√** | **√** | 0.667 |
| Fold 3 | **√** | – | **√** | – | – | **√** | **√** | **√** | 0.833 |
| Fold 4 | – | – | **√** | – | – | – | **√** | – | 0.833 |
| Fold 5 | – | – | **√** | – | – | **√** | **√** | **√** | 1.000 |
| Fold 6 | – | – | **√** | – | – | **√** | **√** | **√** | 0.800 |
| Fold 7 | – | – | **√** | – | – | **√** | **√** | **√** | 0.720 |
| Fold 8 | – | – | **√** | – | – | **√** | **√** | **√** | 0.680 |
| Fold 9 | – | – | **√** | – | – | **√** | **√** | **√** | 0.640 |
| Fold 10 | – | – | **√** | – | – | **√** | **√** | **√** | 0.625 |
| Selected (n/10) | 1/10 | 1/10 | 10/10 | 0/10 | 0/10 | 8/10 | 10/10 | 8/10 | 0.817  (95% CI: 0.696–0.899) |

**Note:** √ indicates the variable was selected by LASSO in that fold; – indicates not selected. Green shading: variables selected in all 10 folds (MCH, Cholinesterase). Blue shading: variables selected in 8/10 folds (PCT, Serum calcium). Orange shading: variables selected in only 1/10 folds (Eosinophil percentage, MCV). Overall cross-validated AUC = 0.817 (95% CI: 0.696–0.899); apparent AUC = 0.842; optimism = 0.025. EOS%, eosinophil percentage; MCV, mean corpuscular volume; MCH, mean corpuscular haemoglobin; EOS (abs), absolute eosinophil count; CRP, C-reactive protein; PCT, procalcitonin; CHE, cholinesterase; Ca, serum calcium; AUC, area under the receiver operating characteristic curve.
